# AI calls the bluff: differentiating benign lesions from triple-negative breast cancer cases

**DOI:** 10.1007/s11547-025-02157-x

**Published:** 2026-01-06

**Authors:** João Mendes, Ana M. Mota, Ana T. Teixeira, Nuno C. Garcia, Nuno Matela

**Affiliations:** 1 Instituto de Biofísica e Engenharia Biomédica, Faculdade de Ciências da Universidade de Lisboa, 1749-016 Lisbon, Portugal; 2 LASIGE, Faculdade de Ciências da Universidade de Lisboa, 1749-016 Lisbon, Portugal; 3 Hospital de Vila Franca de Xira, Estrada Carlos Lima Costa Nº2, 2600-009, Vila Franca de Xira, Lisbon, Portugal

**Keywords:** Artificial intelligence, Breast cancer, Triple-negative breast cancer, Benign lesions, Explainability

## Abstract

Triple-negative breast cancer (TNBC) is the most aggressive molecular subtype of breast cancer (BC). TNBC lacks targeted treatment options, which results in poor clinical outcomes. TNBC lesions usually present benign characteristics on mammograms, complicating their early diagnosis. This retrospective multicenter study presents a convolutional neural network (CNN) model to distinguish TNBC from benign lesions on 566 mammograms (277 benign/289 TNBC), acquired at three different institutions across the UK. Each mammogram had its quality enhanced using a combination of total variation minimization filtering and contrast local adaptive histogram equalization (CLAHE). The proposed model achieved a test set AUC of 0.984, with a sensitivity and specificity of 94.2% and 91.9%, respectively. Explainability with GRAD-CAM was applied to the test set, revealing that the model was using not only lesion characteristics but also tumor microenvironment regions to make predictions. The same test set was analyzed by an expert radiologist who achieved a sensitivity of 71% and a specificity of 60%. The comparison of results between the developed model and the expert radiologist highlights the model’s performance and underscores its potential as a complementary diagnostic tool. This model might help in the task of TNBC early diagnosis, potentially diminishing the number of false negatives.

## Introduction

Breast cancer (BC) is the cancer with the highest number of diagnoses worldwide [1]. The number of women diagnosed with this disease in 2020 was above 2 million [2] and, only in the USA, it is estimated that one in eight women will develop BC in their lifetime [3].

Early BC diagnosis is typically conducted through generalized screening programs with mammography [4]. Despite its positive impacts in terms of mortality reduction [5], some concerns have been raised regarding the occurrence of false diagnoses [6]. Regarding false positives, the risk of having one in Europe is about 20%, while in the USA, 50% of all screened women will experience one [6]. This has a negative impact on women’s mental wellness while also resulting in increased healthcare expenses [6]. In a retrospective study of interval cancers, it was found that nearly 35% were missed in the previous mammogram [7]. The impacts of false-negative results are much more serious than those of a false positive. Missing a timely diagnosis can result in the need for more aggressive treatment and a poorer prognosis.

Invasive breast carcinoma, the most prevalent form of BC, often requires extensive therapeutic interventions. Its characterization largely depends on the associated molecular subtype, which plays a crucial role in determining both disease progression and prognosis. According to immunohistochemical markers—namely hormone receptors (estrogen and progesterone), human epidermal growth factor receptor 2 (HER2) status and Ki-67 levels—this carcinoma is classified into four main subtypes: Luminal A, Luminal B, HER2-enriched and triple-negative [8, 9]. Knowing the molecular subtype is also important to define the treatment plan [10]. Luminal subtypes are hormone receptor-positive and generally have a better prognosis, while HER2-enriched tumors can benefit from targeted therapies. Among all molecular subtypes, triple-negative breast cancer (TNBC) is the most aggressive, with the highest risk of both metastasis and recurrence. Unlike hormone receptor-positive or HER2-enriched subtypes, TNBC lacks targeted treatment options, contributing to its poorer clinical outcomes [8]. For this reason, it is of extreme importance that TNBC is correctly diagnosed at an early stage. However, studies indicate that mammography might be suboptimal for the detection of TNBC as it often lacks typical suspicious mammographic features and may appear as a round mass [11]. In fact, there is evidence that the radiological presentation of TNBC may mimic the appearance of a benign lesion [12].

Given the problems and challenges in screening mammography, especially the occurrence of false negatives, and the lack of malignant features in the early appearance of TNBC [13], there is a clear need for a system that can support clinical decision-making by helping to identify TNBC and distinguishing it from benign lesions. Artificial Intelligence (AI) presents itself as a viable solution to this problem [14]. AI has already been widely used in the field of BC imaging [15], from lesion detection to risk prediction [16]. Particularly, AI has been shown to be capable of increasing the diagnostic accuracy of BC without increasing healthcare professionals’ workload [17].

In this work, we propose the development of a convolutional neural network (CNN)-based method for the differentiation between benign lesions and TNBC tumors in mammography, incorporating explainability measures to better understand the decision-making process.

## Materials and methods

### Dataset and pre-processing

The data used in this study is derived from the OPTIMAM dataset [18], which comprises longitudinal examinations for thousands of women. For this study, we used only the last available diagnostic mammogram and categorized the images into two classes based on the said examinations: benign and TNBC. While TNBC labels were confirmed through pathology reports available in OPTIMAM, benign cases correspond to biopsy-proven benign findings. In total, 566 mammograms were included in our study, consisting of 277 benign cases (48.9%), with an average age of 61.79 years (SD = 8.82) and 289 TNBC cases (51.1%), with an average age of 66.71 years (SD = 6.95).

Our work is a multicenter study using data acquired at three institutions: Jarvis Breast Screening Centre in Guildford, St George’s Hospital in South West London and Addenbrooke’s Hospital in Cambridge.

Each mammogram was cropped to automatically extract a region of interest (ROI) containing the lesion based on the annotations provided in the dataset. All ROIs were then normalized to a pixel value range of 0 to 255 and resized to dimensions of 224*224, aligning with our network’s required input size.

To enhance the quality of the extracted ROIs, three different pre-processing methodologies were implemented and compared to each other: contrast limited adaptive histogram equalization (CLAHE), total variation (TV) minimization denoising and a combination of both. CLAHE [19] aims to improve the contrast of the image by computing several histograms across small sections of an image and performing their equalization. This method differs from common adaptive equalization techniques because it limits the amplification of noise in heterogeneous regions of the image. TV minimization, on the other hand, aims to reduce intensity variations across the image, while preserving sharpness at the edges [20]. The application of these processing methods is standard and automatic, not compromising the potential clinical utility of this method.

### Model: development, training and testing

The backbone architecture used in this study has already proved to be useful in other works by our team, both in the classification of digital breast tomosynthesis [21] and mammograms [22]. In order to tune parameters such as regularization rate (0*.*01 to 1 *×* 10^−6^, in logarithmic steps), pre-processing techniques, and the choice of the optimizer (Adam or stochastic gradient descent (SGD)), a threefold cross-validation approach was employed on the full dataset. After finding the optimal configuration, a final run was conducted by splitting the full dataset into 80% training and 20% testing for evaluation.

Model architecture is deeply described in Table 1. It is composed of four convolutional blocks, with the number of filters increasing as the network deepens (16–32–64–128) and the kernel size diminishing (9–7–5–3). ReLU is used as the activation function. Each convolutional layer is followed by a batch normalization layer that serves the purpose of both promoting convergence and stabilizing the process of learning. The downsampling of the feature maps was done by a max-pooling layer at the end of each convolutional block. After the four blocks, the feature map is flattened and given as input to a fully connected layer with 128 neurons. Classification is done on the final layer, with a sigmoid activation function. Training was conducted during 500 epochs with a batch size of 32. During the threefold cross-validation, done in a patient-independent manner, the mean values of area under the curve (AUC), accuracy, sensitivity and specificity were used to select the best set of parameters. These metrics were then used to assess model performance on the test set.

**Table 1:** Summary of the architecture used. The model is composed by four convolutional blocks followed by batch normalization and max pooling. The feature map is then given to dense layers for binary classification.

Layer type	Output shape	Parameters
Conv2D (9x9, 16 filters)	(216, 216, 16)	1,312
BatchNormalization	(216, 216, 16)	64
MaxPooling2D (2x2)	(108, 108, 16)	0
Conv2D (7x7, 32 filters)	(102, 102, 32)	3,232
BatchNormalization	(102, 102, 32)	128
MaxPooling2D (2x2)	(51, 51, 32)	0
Conv2D (5x5, 64 filters)	(47, 47, 64)	51,264
BatchNormalization	(47, 47, 64)	256
MaxPooling2D (2x2)	(23, 23, 64)	0
Conv2D (3x3, 128 filters)	(21, 21, 128)	73,856
BatchNormalization	(21, 21, 128)	512
MaxPooling2D (2x2)	(10, 10, 128)	0
Flatten	(12800)	0
Dense (128)	(128)	1,632
Dropout (0.5)	(128)	0
Dense (1, sigmoid)	(1)	129

### Explainability

To address the common perception that deep learning models operate as “black boxes,” explainability methods were used in this study. Specifically, we applied gradient-weighted class activation mapping (Grad-CAM) [23]. This method uses gradients to generate visual explanations for the model’s predictions. In the context of this work, the method focused on the activation of the last convolutional layer.

Grad-CAM produces a heatmap, which was overlaid on the corresponding image, highlighting the areas that contributed more to the classification being made. By doing so, we ensure that the model is making logical inferences while it allows us to understand, from a medical perspective, which regions are more indicative of a TNBC diagnosis, in opposition to a benign lesion. This method was applied to the images of the test set which were labeled as “1” (indicating a TNBC diagnosis).

### Clinical validation

To validate the model’s predictions from a clinical perspective, the test set images (without any pre-processing to reflect standard screening assessment) were independently evaluated by an experienced breast radiologist. For each case, the radiologist provided a binary classification (benign or TNBC) without having access to additional clinical or pathological information.

In addition to the classification, the radiologist was asked to assign a confidence score to each decision on a four-point scale, ranging from 1 (low confidence) to 4 (full confidence). This clinical evaluation aims to provide insight into the diagnostic difficulty of each case from a human perspective and to serve as a reference for comparing the model’s performance to that of an experienced clinician.

## Results

### Model optimization

**Fig. 1:**
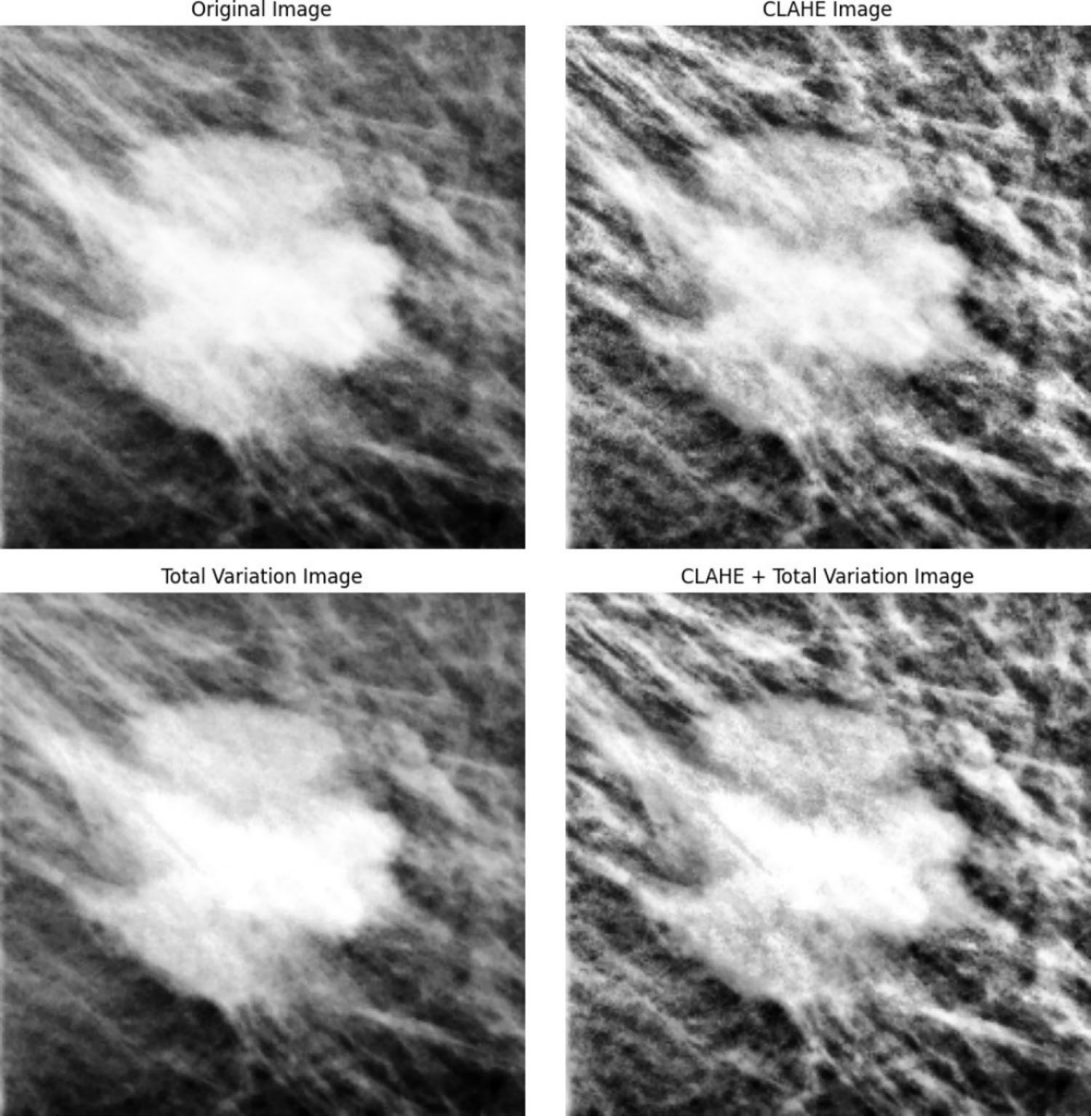
Sample image of the different pre-processing methods. The top-left image represents the original images, while the bottom left depicts the outcome of applying TV minimization to the original image. The top right image shows the result of the application of CLAHE to the original mammogram. Finally, the bottom right image displays the combination of both methodologies.

Figure 1 depicts the effects of the proposed pre-processing techniques on the original image. Applying CLAHE results in a generalized increase in contrast with some texture details on the image becoming more visible. On the other hand, the application of the TV minimization routine to the original image reduces granularity across the image. The filter serves its purpose: fine details are maintained while the entire image appears smoother. Finally, when applying this filter to the CLAHE image there is a clear combination of both methods, with the expected outcome: enhanced texture details with a considerable reduction in granularity (which is particularly noticeable in the tissue surrounding the lesion).

Regarding the cross-validation results, they essentially served the purpose of defining the settings for the final run. The three pre-processing methods were applied and compared. Table 2 depicts the obtained results for each image processing method, using SGD with a learning rate of 1 × 10^−5^.

**Table 2:** Comparison of techniques on performance metrics

Technique	Accuracy	Sensitivity	Specificity	AUC
Original Image	0*.*910 *±* 0*.*019	0*.*925 *±* 0*.*012	0*.*893 *±* 0*.*049	0*.*964 *±* 0*.*012
TV Minimization Image	0*.*911 *±* 0*.*020	0*.*941 *±* 0*.*009	0*.*880 *±* 0*.*048	0*.*967 *±* 0*.*017
CLAHE Image	0*.*926 *±* 0*.*024	0*.*930 *±* 0*.*028	0*.*922 *±* 0*.*025	0*.*969 *±* 0*.*017
TV + CLAHE Image	0*.*926 *±* 0*.*014	0*.*957 *±* 0*.*023	0*.*895 *±* 0*.*029	0*.*975 *±* 0*.*016

Overall, the classification performance was consistently high across all metrics, regardless of the pre-processing technique used. However, the combination of TV and CLAHE stands out among the evaluated methods. While the mean accuracy with this combination is the same as that achieved with CLAHE alone, it demonstrates superior stability, as indicated by a lower standard deviation value. Additionally, the highest AUC value is also observed when combining both techniques, showing the best discriminative capacity between the two classes. However, the highest specificity value is observed for the standalone use of the CLAHE algorithm. The model trained with these images is, in fact, the only one capable of correctly identifying over 90% of the negative class (benign lesions). Nonetheless, the combined use of TV and CLAHE results in the highest specificity value, with over 95% of the positive instances being correctly classified. Considering the clinical relevance of the proposed task, misclassifying a TNBC case and considering it a benign lesion results in more negative consequences than the other way around. For that reason, it is clinically more relevant to prioritize a model with a higher ability to correctly identify positive cases. Given that, the combination of both pre-processing methodologies was chosen to be used on the final run of the model. Despite the relatively small dataset, the high performance metrics and the very small standard deviations of the mean values provide confidence that the model is robust and capable of generalizing to different imaging sets.

### Model validation

On this final run, the entire dataset was divided into training (80%) and testing (20%) and the images were pre-processed with both TV minimization and CLAHE.

**Fig. 2:**
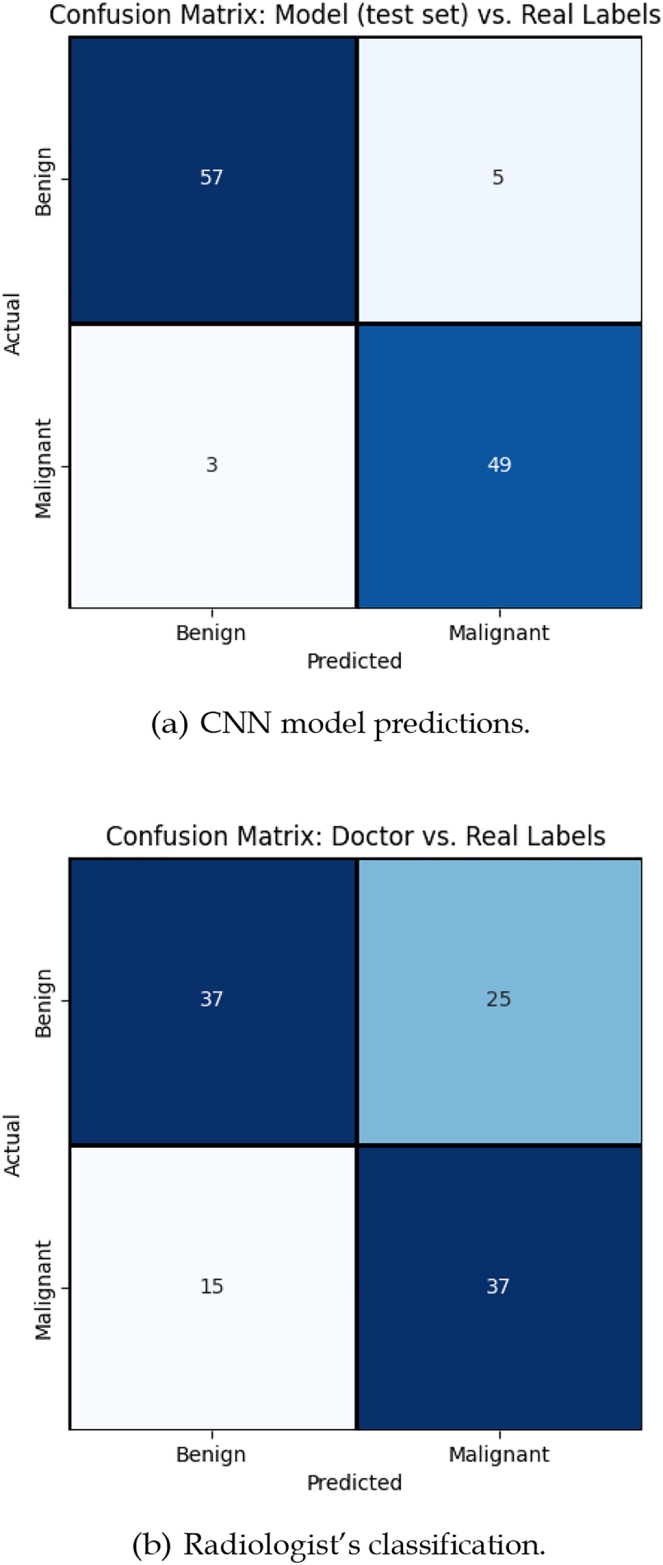
Confusion matrices on the test set: **a** CNN model predictions, **b** Radiologist’s classification.

The test set results are in line with what was seen for cross-validation. The performance metrics obtained on the test set are shown in Table 3 and Fig. 2a depicts a confusion matrix also obtained when feeding the test set to the model.

**Table 3:** Performance metrics computed on the test set

Metric	Value
Accuracy	0.930
Sensitivity	0.942
Specificity	0.919
AUC	0.984

As shown in Table 3, the results align with expectations. Sensitivity remains very high, while specificity now exceeds 90%. Additionally, the AUC achieved in this final evaluation is higher than the average AUC obtained during the threefold cross-validation. Overall, nearly 93% of the cases, despite their class, were correctly classified. Out of the 51 TNBC cases, only three of them were misclassified as benign. The number of cases that failed on the benign class was slightly higher (5), which can be perceived by the sensitivity and specificity values. As stated before, correctly identifying both TNBC and benign cases is important; however, detecting TNBC is particularly critical due to its clinical implications. The developed model addresses this by maintaining a very low number of false-negative results.

### Radiologist’ analysis

Figure 2b depicts the confusion matrix of the classification done by the radiologist. In total, 65% of the cases are correctly classified (37 benign and 37 malignant). The radiologist tends to make more errors when classifying benign cases, with 25 misclassifications. Summarizing, the radiologist presented a sensitivity of *≈* 71% and a specificity of *≈* 60%. Table 4 presents the radiologist’s average confidence levels for both correct and incorrect classifications. The highest confidence is observed in correctly classified malignant cases, whereas the lowest confidence appears in cases where benign cases were incorrectly classified as malignant—also the most frequent type of error. This seems to indicate that, when in doubt, the doctor tends to adopt the conservative approach of classifying the case as malignant.

**Table 4:** Mean and standard deviation of the confidence on the classification made by the radiologist.

	Predicted	
Real Label	Benign	Malignant
Benign	3.14±0.89	2.92±1.12
Malignant	3.27±0.80	3.59±0.69

### Model explainability

**Fig. 3:**
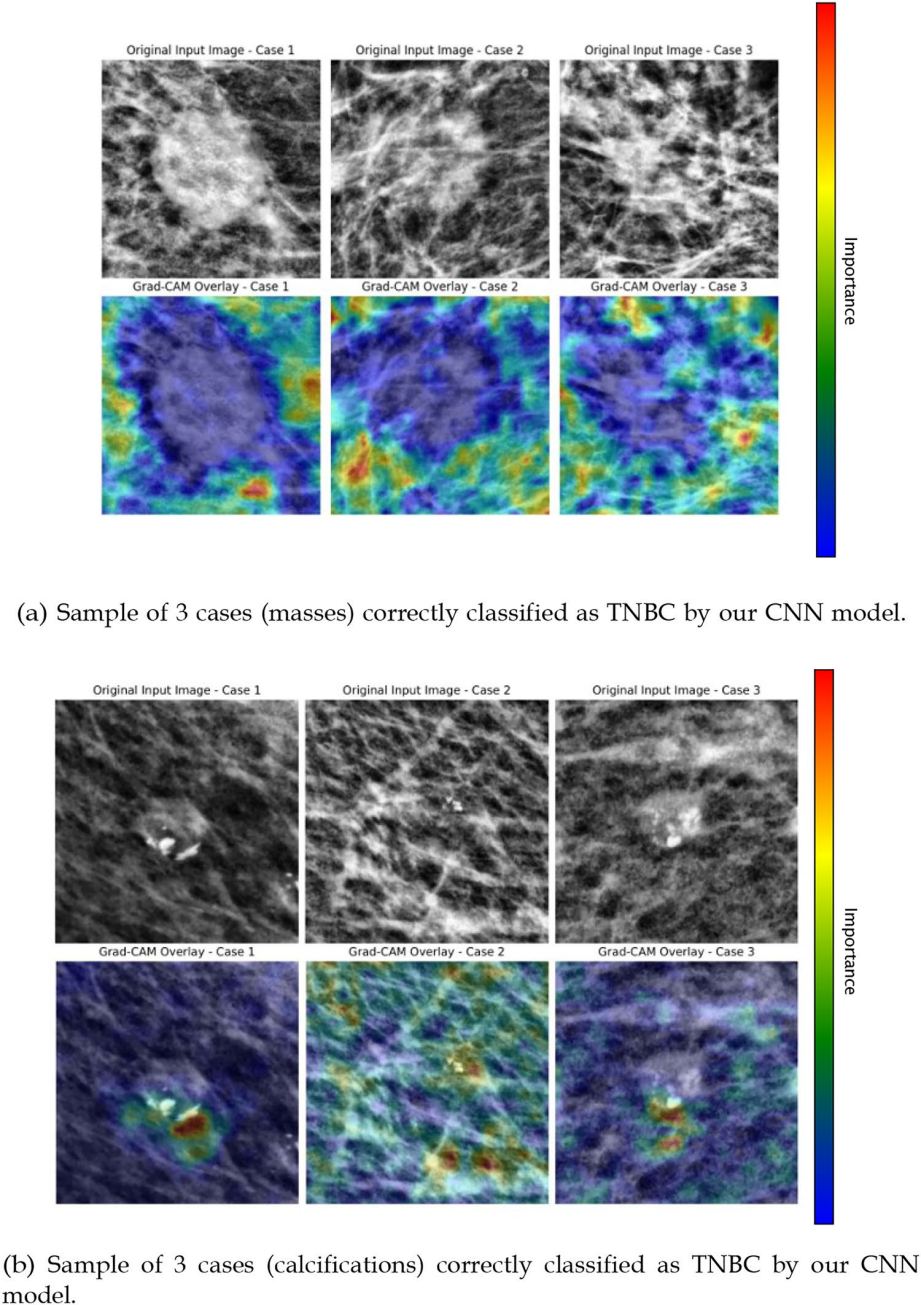
Representative examples of triple-^1−0^negative breast cancer (TNBC) cases correctly classified by the proposed CNN model: **a** masses, **b** calcifications

Regarding the explainability results, a figure composed of three different TNBC mass cases from our test is presented (Fig. 3a). Figure 3b shows three TNBC calcification cases, correctly classified by our network. These figures show the original image and the respective Grad-CAM heatmap overlay—with the red areas representing the locations that contributed most to the classification. As the colors on the heatmap transition to blue, those locations on the image contribute less to the classification.

**Fig. 4:**
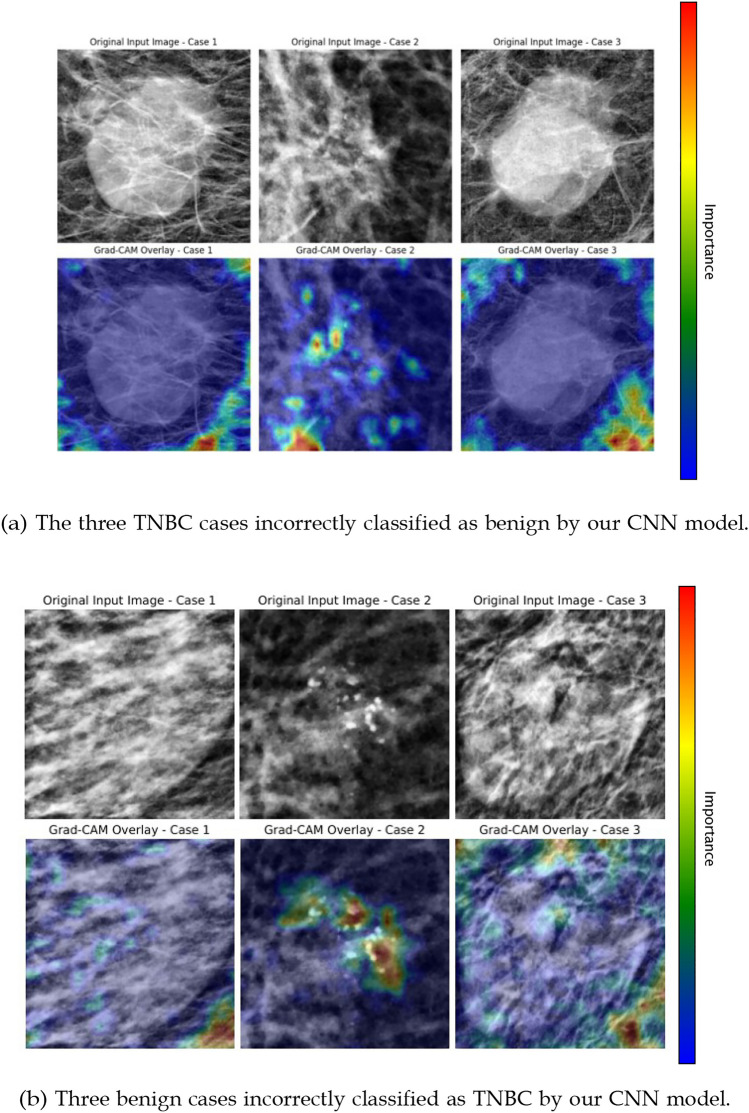
Misclassified cases by the proposed CNN model: **a** TNBC cases predicted as benign, **b** benign cases predicted as TNBC

Figure 4a, on the other hand, depicts the three TNBC cases that the model failed to classify, alongside their respective heatmaps. Three benign cases identified by our model as being TNBC are depicted in Figure 4b

## Discussion

The main goal of this work was to develop an AI-based model capable of analyzing mammogram ROIs containing lesions and differentiating TNBC lesions from benign lesions. A threefold cross-validation methodology was employed in order to select the best parameter setting. The use of SGD optimization with a learning rate of 1 × 10^−5^, and the combination of two pre-processing methods (TV minimization and CLAHE) were selected for the final run of the model.

In total, our test set comprised 114 images, of which 106 were correctly predicted as belonging to their real class. This results in an accuracy of nearly 93%. Moreover, only 3 TNBC cases were incorrectly allocated to the benign class, while for the benign cases, only five of them were misclassified. These facts resulted in a sensitivity of nearly 94% and a specificity of almost 92%. The robustness of the performance metrics with respect to data variability is supported by the cross-validation results, where all metrics exceeded 89% and exhibited standard deviations below 3%.

One of the key strengths of the developed model is its high sensitivity. In a clinical context, the mammographic appearance of TNBC might not present features that are characteristic of malignancy (size, irregular shape, spiculated margins) and therefore result in a misdiagnosis. Despite such variability, the developed model is capable of correctly classifying 93% of all cases. This suggests that our model may contribute to reducing the risk of misdiagnosis, thereby helping to avoid delayed detection and improving patient prognosis. Moreover, the very high specificity value indicates a strong capability of identifying the benign cases, which can contribute to diminishing the probability of false-positive results that can lead to unnecessary follow-up procedures and increased healthcare costs.

Nonetheless, the presented model goes beyond making predictions, as efforts were made to ensure it does not operate as a mere black-box. We incorporated Grad-CAM in our pipeline to provide visual and clear explanations of the predictions made by the model. The results suggest that, beyond the lesion itself, the differentiation between the two classes is largely influenced by the surrounding tissue. These findings are in line with the state-of-the-art, which suggests that the surrounding microenvironment contains valuable information that contributes to the evaluation of breast lesions [24]. These findings are also clinically meaningful. The main difference between a malignant tumor, such as TNBC, and a benign lesion, lies in the malignant tumor’s ability to invade and spread to surrounding tissues. Given this, in scenarios where the lesion itself does not display clear signs of malignancy, the model naturally focuses on the characteristics of the surrounding tissue to differentiate between classes. The highlighted regions often correspond to subtle architectural distortion, asymmetry of parenchymal texture or localized density variations surrounding the lesion—findings that may reflect stromal reaction or desmoplastic changes characteristic of TNBC. This behavior suggests that the model is effectively learning to identify subtle tissue modifications that are indicative of malignancy, even when such changes are not easily distinguishable to the human eye and the tumor itself appears less distinctive. Notably, when the TNBC lesions are masses, the model focuses only on the surrounding tissue (Fig. 3a); however, when the lesions are calcifications (as seen in Fig. 3b), the model’s prediction mainly relies on the lesion itself.

The comparison between the CNN and the expert radiologist revealed informative patterns, particularly when analyzed alongside the reported confidence levels. In general, the radiologist’s correct classifications corresponded to cases with higher confidence and lower standard deviation, indicating consistent and assured decisions. However, in the 15 cases where malignant lesions were incorrectly classified as benign by the radiologist, the average confidence level was high and the standard deviation was low. This suggests a systematic and generalized misperception, which is clinically concerning in the context of TNBC diagnosis. In contrast, when benign lesions were misclassified as malignant, the associated confidence levels were lower and more variable: an indication of diagnostic uncertainty that, while resulting in false positives, may be preferable in clinical settings to avoid missed malignancies.

Further analysis of discordant cases between the model and the radiologist provides additional insights. Among the 62 benign cases, five were misclassified by the CNN as malignant. Of these, two were also classified as malignant by the radiologist (with confidence levels of 3 and 4), while the remaining three were correctly identified as benign, though with moderate confidence (confidence levels of 2, 2 and 4). Conversely, among the 52 malignant cases, three were misclassified by the CNN as benign. One of these was also incorrectly classified as benign by the radiologist (confidence level 3), whereas the other two were correctly identified as malignant with full confidence (confidence level 4). Figure 4a illustrates the three malignant instances where our model failed. The radiologist accurately classified “case 1” and “case 3,” identifying patterns that classified the tumor as malignant. However, our model failed to find meaningful information on these cases, as shown by the inconsistency in the Grad-CAM overlay (opposite to what happens on the correctly classified cases seen in Fig. 3a).

The comparison between the CNN and the expert radiologist highlights the complementary nature of human expertise and artificial intelligence. While the model demonstrated superior performance, there were clinically relevant cases where the radiologist outperformed the model, correctly identifying malignant lesions that were misclassified by the CNN. These findings reinforce the idea that AI models should not replace clinicians, but rather serve as supportive tools, particularly in complex or borderline cases.

Despite the promising results, the study presents several limitations. The dataset used was primarily acquired from a single imaging device and is limited to the geographical regions where the participating sites operate. Gathering data from different sources is important since it allows to gain a wide-view of the true variability of the clinical presentation of both TNBC and benign lesions. Moreover, the amount of data is also limited (< 600 instances) and future work might benefit from data augmentation techniques, specifically with the use of generative adversarial networks. However, the use of data augmentation in this scenario needs to be even more cautious. The goal of the model is to differentiate two types of lesions that have a very close mammographic appearance, in opposition to a simple benign/malignant classification. The development of synthetically created lesions should be clinically validated before their incorporation in a future study. Nonetheless, in order to avoid any potential bias due to the small nature of the dataset, cross-validation was performed to assess model’s performance across different image sets. The obtained results show not only very high values for every metric, as the dispersion metric values are very small (see Table 2). These results give confidence in the robustness and generalizability of the model. Another limitation is the relatively simple explainability method, future efforts should focus on increasing the interpretability of the model, giving more relevant information to clinicians rather than a standalone heatmap. The future inclusion of techniques, such as SHapley Additive exPlanations (SHAP), could contribute to a better explanation of the model once it allows the user to understand what features are impacting classification, either positively or negatively. Nonetheless, the saliency maps produced by our GRAD-CAM approach already substantially contribute to a better explainability of the model. Instead of just giving a final classification to the users, the model provides a salience map for each instance, indicating which regions of the image are contributing to the classification. This promotes a better understanding of the model and increases AI trustworthy.

Another important limitation of work is the reliance on a single radiologist for mammogram assessment. Even though the radiologist involved has several years of experience and analyses mammograms regularly, this approach does not account for interobserver variability. Due to different trainings, reading styles, and years of experience, radiologists might have different perceptions regarding the classification of a lesion. By having only one radiologist evaluating the lesions and comparing them to the model, it might result in an overestimation of the model's capability in relation to the clinicians. Moreover, the radiologist’s lack of access to prior mammograms may have further contributed to an underestimation of clinical performance compared to the AI model. Access to previous exams can be crucial for detecting subtle temporal changes and making more accurate diagnostic assessments. Incorporating several radiologists in future studies would provide a more robust clinical assessment of the mammograms, removing individual bias from the classification, and allowing fairer comparisons with the developed AI model.

The need for a ROI definition before giving the mammogram to the model could be considered a limitation of this work in the sense that it could increase radiologists’ work burden. However, the proposed task does not simply aim to analyze a mammogram and determine whether a lesion is benign or malignant, as is typically the primary concern in a screening context. The task proposed here is more specific and concerns the differentiation of two types of lesions that commonly have the same mammographic appearance. Given that, in a real-world context, this model would be applied after the lesion is found by clinicians and would serve as a second opinion on a differential diagnosis.

Despite these limitations, the developed model demonstrates the potential that AI has in the differentiation of TNBC from benign lesions, presenting itself as a possible aiding tool to increase diagnostic accuracy and alleviate the burden of BC. Moreover, this model has the potential to be especially valuable in assisting junior radiologists who are still in the early stages of their clinical careers and may lack the experience to confidently interpret more challenging cases.

## Conclusion

This study proposed a CNN model that distinguishes TNBC from benign lesions in mammograms. The model demonstrated high sensitivity and specificity, indicating a strong ability to identify both malignant and benign cases. Explainability methods were incorporated in the pipeline, serving as support for the users to understand what information from the mammograms is being used for predictions. Future work should focus on overcoming the referred limitations: using an entire mammogram instead of an ROI, increasing data in terms of absolute number of instances and in terms of variability and searching for deeper explainability measures.

## Declarations

### Data access and governance

Access to OPTIMAM data was granted following a formal application process evaluated by the OPTIMAM Data Access Committee.
